# Piloting Patient Reported Outcome Measures in Cleft Lip and Palate Services in Aotearoa New Zealand

**DOI:** 10.1177/10556656261431254

**Published:** 2026-04-15

**Authors:** Kenny Ardouin, Nicola Stock, Tika Ormond, Phoebe Macrae

**Affiliations:** 1 School of Psychology, Speech & Hearing, 2496 University of Canterbury, Christchurch, New Zealand; 2 Centre for Appearance Research, University of the West of England, Bristol, UK

**Keywords:** patient satisfaction, psychosocial adjustment, quality of life, speech therapy, young adults

## Abstract

**Objective:**

Standardized patient-reported outcome measures (PROMs) are rarely used in cleft lip and palate (CL/P) care within New Zealand (NZ). This pilot study sought to obtain a baseline of patient-reported outcomes in NZ and evaluate clinicians’ experiences of implementing PROMs into CL/P services.

**Design:**

A mixed methods cross-sectional design using CLEFT-Q scales. Data were compared to published CL/P CLEFT-Q norms using *t*-tests, and within-group differences were investigated. Qualitative interviews were analyzed using codebook thematic analysis.

**Setting:**

Five CL/P teams in Aotearoa New Zealand.

**Participants:**

Patients aged 12+ years (*n* = 101) were recruited through the five Health NZ CL/P teams between April 2023-March 2024. Five CL/P team members responsible for recruitment participated in an interview at the conclusion of the trial.

**Results:**

Compared with normative data, patients reported more favorable outcomes on speech distress and school function, and poorer psychological function. Speech and social function scores were aligned with CL/P norms. Patients aged 18+ reported the lowest psychological functioning and females reported the lowest social functioning. Clinicians recognized the value of PROMs, yet highlighted concerns about insufficient resourcing to administer, review, and provide follow-up.

**Conclusions:**

Psychological function is indicated as an area of concern, particularly for adults with CL/P. Routine use of PROMs would evaluate change in measures of function over time and enhance patient-centered holistic care. Greater personnel and IT resourcing would facilitate routine implementation of PROMs in CL/P teams.

## Introduction

### Patient Reported Outcome Measures in CL/P Care and Research

Understanding the patient perspective and incorporating patient voice into treatment plans and decision making is essential to the delivery of patient-centered care^1,2^ and can lead to better health outcomes.^3^ Patient-reported outcome measures (PROMs) can be used to understand the perspectives, concerns, and priorities of the patient and recognizing patients as experts in their own experiences.^4–6^

PROMs are widely-used in broader healthcare, particularly in areas such as mental health,^7^ and cancer care.^8^ PROMs are recognized as a valuable tool to enhance patient-centered care, shared decision making, and patient engagement.^9,10^ PROMs can also provide the clinician with insights about their patient and their perspective, which they otherwise would not have.^10^ Although CL/P-specific PROMs have existed for over a decade, their use as a clinical assessment and evaluation tool is not uniform.

CL/P-specific PROMs that have been developed for and are used in CL/P clinical practice and research include tools such as the Cleft Hearing, Appearance and Speech Questionnaire (CHASQ),^11,12^ the PAT-CV, and the CLEFT-Q.^13^ Additionally, generic measures (eg, the Strengths and Difficulties Questionnaire; SDQ, PROMIS, Pediatric Quality of Life Inventory; PedsQL)^14^ are often used in CL/P care and research allowing for comparison of the CL/P population to the general population, or across conditions.^5^ Several comprehensive assessment packages which contain both objective assessment tools and PROM measures are already in use internationally.^15,16^ Examples include the International Consortium for Health Outcomes Measurement (ICHOM) assessment protocols (ICHOM Sets), which include a set for CL/P,^17,18^ the UK consensus measures,^16^ and the Americleft Collective Recommended Measures which includes both CL/P specific measures (such as CLEFT-Q) in addition to general measures (such as PedsQL).

### CL/P Prevalence and Service Provision in New Zealand

The prevalence of CL/P in NZ is slightly higher than the global average, affecting approximately 1 in 560 live births, with indigenous Māori having the highest incidence of cleft palate (∼1 in 460).^19^ Treatment is provided through one of five regional multidisciplinary CL/P teams. A notable discrepancy of NZ's CL/P services is that there is limited clinical psychology provision with 4 of the 5 teams having no psychology provision, and the other team having a psychologist employed for just 0.2 FTE (20%).^20^ Although services in NZ recognize CL/P as a lifelong condition, there is currently no service specification that ensures adults can access ongoing CL/P care.^21^ This has led to a “postcode lottery” where people may, or may not, be able to access psychological care depending on where they live.^22^

### Existing PROM use in New Zealand CL/P Teams

Although the NZ government Health Quality & Safety Commission use PROMs to evaluate health system effectiveness, quality improvement, patient-centeredness, value, and equity,^23^ there is no current protocol in NZ which specifies the use of PROMs within CL/P services, and existing CL/P PROMs have not been standardized on the NZ population. However, two CL/P teams have developed their own tools to incorporate patient reported outcomes into practice which are being used routinely, one using a caregiver-reported “Smiley Face” PROM,^24^ and the other a patient-reported psychological screener (the score is used to determine whether a patient will be seen by the team's psychologist). Notably, neither tool has been psychometrically validated, and the latter has not been made publicly available. The three other CL/P teams in NZ reported not currently using any PROM measures within routine clinical care.

Inconsistent use of PROM measures, and lack of standardization throughout NZ makes it difficult to understand patient-reported outcomes nationally. Furthermore, without using PROMs with normative data, it is unclear where New Zealand CL/P patient-reported outcomes sit relative to other countries. Finally, existing use of caregiver-reported outcome measures are valuable but cannot be assumed to be a proxy for patient-reporting given known discrepancies between patient- and parent-reported data.^25,26^

### Aims

This study sought to answer two research questions by piloting the implementation of a standardized PROM into hospital-based CL/P services and evaluating clinician perspectives of implementing the trial.

1. To understand current patient satisfaction in the domains of speech function and distress, psychological, social, and school functioning (using the CLEFT-Q PROMs) in NZ.
2. To understand CL/P clinician attitudes toward and experiences of PROMs and the CLEFT-Q PROM NZ pilot.

## Methods

### Design

This study utilized an empirical mixed methods design. Ethical approval was obtained from the New Zealand Government Health and Disability Ethics Committee (HDEC), approval number 2022/13375. Māori consultation approval was obtained from Ngāi Tahu Consultation and Engagement Group (Te Wai Pounamu - South Island), Waikato Research Advisory Group (Waikato), and Regional Advisory Group-Māori (Pōneke - Wellington). Finally, Locality Assessment to conduct the research in the five CL/P teams around NZ was approved from the respective committees at Middlemore, Waikato, Hutt, Christchurch, and Dunedin Hospitals.

### Aim One

To address the first aim, standardized patient-reported outcome measures were administered via an online survey over a 12-month period to assess speech distress, and speech, psychological, social, and school function. Measures were administered by clinical staff across five Health New Zealand CL/P teams. Participants could either complete the measures within the waiting room, or online at home.

#### PROM Selection and Licensing

The CLEFT-Q was selected given its specificity for CL/P, comprehensive CL/P normative data (normed from age 8, and with a considerable international dataset) and psychometric validation, ease of availability, and as each of its scales can be administered independently of each other.^24^ A license to use the CLEFT-Q patient outcome measures for this research was obtained from McMaster University.

#### Participants

Eligible patient participants were anyone aged 12+ (including adults) who attended a CL/P clinic appointment or speech language therapy appointment at any of the five NZ CL/P teams during the study period (April 1, 2023–March 31, 2024), or had been seen by their CL/P team within the last 2 years. Eligible participants were provided a Qualtrics link by their CL/P team to complete the survey online. Alternatively, patients meeting the criteria could access the survey from the Cleft NZ Facebook page (support organization). The minimum age of 12 (as opposed to 8 which the CLEFT-Q is normed down to) was selected to increase the probability that collected data reflected patient perspective, rather than being unduly influenced by parent perspective. Despite these efforts, it is acknowledged that patients may discuss their responses with their parents; a natural occurrence that is tricky to eliminate in research and clinical settings. Recruitment methods were tracked to identify how participants had accessed the study. Participants were instructed to only complete the survey once and generated a unique participant code from their name and date of birth to prevent duplicate entries whilst maintaining participant anonymity from the research team.

#### Data Collection

Eligible participants were provided with an information sheet by their clinician or the clinical administration team either in-person or via (e)mail. Once patients had read through the information, they could either complete the survey on their own device by scanning the QR code or clicking a link, or by using a University of Canterbury-issued tablet (if attending an in-person appointment). In all cases, participants would complete an online consent form, and then would proceed to generate their participant code, provide demographic information, and then complete the CLEFT-Q PROMs. The CLEFT-Q scales that were used in this study were the speech function, speech distress, psychological, social, and school function scales. The school function scale could be skipped entirely if participants were no longer attending school. The demographic questions asked of all patient participants are presented in supplementary materials.

Once survey data were submitted, it would trigger two emails—one to the research team for data analysis, and another to the Coordinating Investigator at the CL/P team site containing the survey data. The format of the participant codes allowed CL/P team staff to identify the participant, while ensuring the research team was unable to do so. As a result, the email to the Coordinating Investigator was able to be used as part of the patient's clinical consultation if the CL/P team chose to do so. Participants were fully informed and agreed to this use of their data when completing the consent form.

All responses were reviewed by the research team clinician within 24 hours of submission. Any low scores were discussed with the relevant Coordinating Investigator who had the means to deidentify the participant code with participants’ permission. The Coordinating Investigator would then determine whether follow up was required: this typically included the clinical team contacting the patient to discuss the identified areas of concern to gain additional context and either see the patient themselves or refer to colleagues within the CL/P team. Additionally, the University of Canterbury's clinical psychologists were able to offer free intervention to participants within the study with urgent unmet psychological needs if necessary.

#### Data Analysis

For each participant's data, a sum score was first calculated for each scale using the scoring manual.^13^ Once the sum score was calculated, a Rasch score was derived using the conversion table within the scoring manual. Rasch scores for each scale were then entered into IBM SPSS statistical software (Version 30.0.0.0 (172)) alongside reported demographic information.

Shapiro-Wilk normality testing and Q-Q plots were completed to assess the collected sample for normality. Nonparametric one-sample Wilcoxon signed-rank testing was carried out to compare the sample dataset to the normative data.

Following comparison of the study cohort to the international CL/P normative data, post hoc supplementary analyses were run to identify any differences between demographic variables of age, gender, ethnicity, and cleft type on the speech function, distress, psychological, social and school function scales. These analyses utilized Independent-Samples Mann-Whitney U Tests for demographic variables with just two groups (ie, age and gender), while Independent-Samples Kruskal-Wallis tests were used for demographic variables with three or more groups (ie, ethnicity and cleft type).

### Aim Two

To address Aim 2, semistructured qualitative interviews were carried out with CL/P team staff who were administering the study materials and were audiorecorded and transcribed verbatim.

#### Participants

Eligible clinician participants were defined as the clinical staff from each CL/P team who had been the named Coordinating Investigator on their region's locality assessment application and/or their nominated delegate. These clinicians were eligible due to their direct involvement with implementing PROMs within their team.

#### Data Collection and Analysis

Qualitative data consisted of semi-structured interviews with the clinicians who were responsible for implementing the PROMs study within their hospital. Interviews took place at the conclusion of the PROMs pilot over Zoom between March and April 2024. Clinician participants completed a consent form, and all meetings were audio recorded using a digital voice recorder and Zoom cloud recording. Participants were reminded to maintain patient confidentiality. Interview questions were developed to elicit broad discussion on the PROMs pilot and gain a pragmatic understanding of the impact of PROM administration on clinical practice, and perceived facilitators and barriers to routine PROM use. The interview questions and prompts are provided in supplementary materials. Although these questions were used to initiate discussions, clinicians were reminded that they were free to discuss pertinent perspectives and information as they desired. Interviews were facilitated by the lead researcher and lasted between 30 and 44 min in duration (Mean = 40 min).

Using Otter.ai Transcription Software, interviews were transcribed verbatim. The lead researcher reviewed and edited the AI-generated transcriptions to ensure consistency with the audio recordings.

The dataset was analyzed qualitatively using inductive codebook thematic analysis.^27^ Using an inductive approach allows for a data-driven approach where the codebook (and resultant themes) are derived from the data without preconceived codes or themes. Following transcription, the data were re-read by the first author and quotes of interest were identified. Quotes were then coded by the first author, with similar quotes ascribed the same code. Once initial coding was complete, codes were discussed and refined by the research team. Similar codes were grouped together to form themes.

## Results

### Aim One

One hundred and one patient participants contributed data to this study. Of these, all 101 completed psychological function, and social function scales, 88 completed speech function, and speech distress scales, and 69 completed the school function scale. Participants’ demographic data are presented in Table 1.

**Table 1.** Patient Participant Demographics.

	Number of participants (*n*)	Percentage
*Cleft Team region*		
Auckland	43	42.6
Hamilton	1	1.0
Wellington	26	25.7
Christchurch	21	20.8
Dunedin	8	7.9
Online (no Cleft Team)	2	2.0
*Age*		
12-17	60	59.4
18+	41	40.6
*Gender*		
Female	58	57.4
Male	42	41.6
Describe in another way^	1	1.0
*Ethnicity*		
NZ European	60	59.4
Māori	13	12.9
Pacific Peoples	8	7.9
Asian	10	9.9
Other European	5	5.0
Unknown	5	5.0
*Cleft Type*		
UCLP	36	35.6
BCLP	13	12.9
CPO	35	34.7
UCL^#^	6	5.9
BCL^#^	2	2.0
Other Cleft Type	3	3.0
Unknown	6	5.9

^This person was excluded from within-group gender analyses; however, their data was included in all other analyses.

^#^These two groups were combined to form a CLO group for within-group analyses.

Additionally, 13 participants completed appearance of the face, teeth, and jaws scales. This sample size was considered insufficient to draw meaningful conclusions, and these scales were subsequently removed from the dataset.

#### Patient-Reported Comparisons With Normative CL/P Data

Shapiro-Wilk normality testing found that none of the scales were normally distributed within the sample dataset. Q-Q plots revealed clear deviation at the extremes, most notably a high number of values at the maximum of the scale. This feature does not materially affect the mean or the median, however, does disrupt the shape required for a normal distribution. Therefore, non-parametric testing (one-sample Wilcoxon signed-rank test) was selected to compare this dataset to the published norms in lieu of a one-sample *t*-test.

The study sample mean, median and standard deviation, and the normative sample means and standard deviation for each CLEFT-Q scale used in this pilot are presented in Table 2 alongside the results of the one-sample Wilcoxon signed-rank tests.

**Table 2.** Comparison of CLEFT-Q Scores to International CL/P Normative Values.

CLEFT-Q Scale	NZ Sample Mean [Median] (SD) *n* = X	Normative Sample^28^ Mean (SD) *n* = X	One-sample Wilcoxon signed-rank test normative data to NZ data
Speech Function	68.44 [69] (23.03) *n* = 88	69.30 (21.30) *n* = 1869	*p* = 0.824
Speech Distress	77.99 [77] (20.56) *n* = 88	69.10 (21.30) *n* = 1869	*p* = 0.000*
Psychological Function	68.50 [66] (21.66) *n* = 101	73.80 (18.90) *n* = 2255	*p* = 0.021*
Social Function	72.75 [68] (19.25) *n* = 101	73.10 (17.30) *n* = 2263	*p* = 0.999
School Function	80.99 [79] (17.97) *n* = 69	75.90 (17.70) *n* = 1663	*p* = 0.007*

* That finding is statistically significant (*p* < 0.05).

No differences in speech or social function were observed between the current pilot study data and international CL/P normative data. Significantly more favorable speech distress and school function scores were found compared to the international CL/P normative sample. However, significantly poorer psychological function scores were reported compared to international CL/P normative data.

#### Within Group Comparisons by Demographic

Data from the speech function, speech distress, psychological, social, and school function scales were analyzed for within group differences. Responses from two participants with BCL (n = 2) were merged with the UCL group to form a Cleft Lip Only (CLO) group. Finally, one participant did not identify as male or female and were therefore removed from the analyses by gender, although their data were included for all other analyses.

#### Influence of Cleft Type and Ethnicity

Independent samples Kruskal-Wallis analyses found no significant differences on any measure by cleft type or ethnicity.

#### Influence of Age

Independent samples Mann-Whitney U testing found there were statistically significant differences by age on the psychological and social function scales. These analyses found that psychological function was lower in the 18 + group than the 12–17 year old group (mean of 62.68 vs 72.47; *p* = 0.030). Similarly, the 18 + group reported poorer social function than the younger age group (mean of 68.98 vs 75.33; *p* = 0.040).

#### Influence of Gender

There were no differences in speech function, distress, or psychological, and school function by gender, however on the social function scale, females reported lower scores on this measure relative to males (mean of 69.88 vs 77.38; *p* = 0.050).

A summary of results of the within group analyses are available in supplementary materials.

### Aim Two

Five clinicians from four CL/P units across NZ chose to participate in interviews at the end of the study. Of these, four were Speech Language Therapists, and one was a Cleft Coordinator.

Three themes were identified using codebook thematic analysis: (1) pre-existing attitudes and practices to incorporating patient perspective within cleft care, (2) perceived influence of routine PROMs use within cleft clinics, and (3) facilitators and barriers to introducing PROMS routinely within cleft teams.

Summaries of each theme with illustrative quotes are presented below, with detailed codes and additional exemplar participant quotes provided in supplementary materials. Shortened quotations include (…), and additions represented by [xxx] for clarification. Participants collectively referred to as “all” include all or all but one, “most” include more than half, and “some” include less than half as per qualitative data reporting conventions described by Hill et al.^29^

#### Theme One: Pre-Existing Attitudes and Practices to Incorporating Patient Perspective Within Cleft Care

All clinician participants reported feeling proud of their involvement with the PROMs pilot, although the data also shows that in most cases, they encountered challenges or pushback to PROMs implementation within their team. For example, one participant reported:

“I was very enthusiastic at the beginning. I couldn’t believe how unenthusiastic my team were…I think this research here is very needed.”

There were a range of existing practices in place for incorporating patient voice into clinical care prior to the PROMs pilot, with some teams having developed their own screener or PROM, while others used discipline-specific PROMs such as the RhinoCleft^30^ within speech language therapy.

“We have [our own PROM] which has 10 or so questions to say what people think of smile, face, social interactions, various things … that gives us a bit of an idea of what are the things that they feel need to be addressed.”

Some teams viewed clinical consultations as the method by which to obtain patient input into their care.

“Discussion in the clinic would be the usual way … their [patient] input would be sought in that way.”

Clinician participants reported their personal support of PROMs implementation, but that they had encountered a diversity of perspectives within their teams to using PROMs, including some who were resistant to PROMs or questioned the efficacy of gathering patient perspective using such a tool. There was repeated mention that PROMs can feel like an audit tool and that there was skepticism of external auditing. Nevertheless, clinician participants did not feel these were valid reasons not to persevere with introducing PROMs, as they recognized the benefits to patients and the service by documenting patient perspectives.

“A general culture within a team or an attitude shift needs to occur … no one likes audits … but I think outcome measures are so important.”

However, despite support of PROMs by all clinicians in this study, no teams used the data clinically, and those who were using self-developed screeners indicated a preference to continue to use these rather than a validated PROM, such as the CLEFT-Q.

“Our [own PROM] works well for us. And we will continue to use them until we find another way of getting that easy access to information at the time. It might not be the best thing but it works.”

#### Theme Two: Perceived Influence of Routine PROMs Use Within Cleft Clinics

Clinician participants reflected on perceived benefits of routine PROM implementation. These included that PROMs could be a means to check whether patients, family, and clinicians are aligned regarding treatment goals, and helping to orient patients to the purpose of the visit. Conversely, if PROMs showed misalignment between parties, they could serve as a valuable tool to identify where discrepancies exist and facilitate further discussion.

“It could help to inform for the child, the reason why the parent keeps dragging the child to the appointment all the time as well … Why do we care? Why do we want you to keep coming back? Why do we want to help you? Do you think you need help?”

Clinician participants identified the utility of PROMs as a way of asking and answering difficult questions without needing to do so in a face-to-face consult.

“Perhaps they might get more time to consider a more sort of considered and honest answer to things [when using PROMs]. Whereas in the middle of an appointment where there's lots of professionals in the room … perhaps they fear taking up too much time or not saying the right thing.”

Some clinician participants also discussed how PROMs could be a prompt for patients to share concerns and ask questions, recognizing that patients can oftentimes forget to ask pertinent questions once they enter the consultation room. One participant considered PROMs could facilitate equitable care by ensuring the same questions are asked of all patients, rather than skipping questions in situations where the clinician believes they already know the answer.

“Does it [use of PROMs] support equality of care, because we’re ensuring that the same questions are asked to everyone?”

Some clinician participants also stated that PROM use could give them enhanced confidence in the wishes of patients and their families.

“I think it will be a really helpful thing to be able to get their patient voice and get what's on top for them, because it might not be what we’re looking at.”

Most clinician participants relayed concern that routine PROMs implementation could have a negative influence by introducing concerns for patients which were not already present, and/or that PROMs may identify concerns which the CL/P team is not resourced to remedy.

“We’re getting them to highlight problems when there really isn’t a problem. That is one of the reasons that they [colleagues] have told us that they don’t think patients should be reporting outcomes.”

Furthermore, some participants relayed concerns from other team members that patients may worry about how their PROM responses may impact upon future treatment and/or relationships with clinicians.

“The concern [with using PROMs clinically] is that it might bring hope to people that something can be fixed, when in fact, the surgeon knows that due to surgical limitations it can’t be.”

#### Theme Three: Facilitators and Barriers to Introducing PROMs Routinely Within Cleft Teams

Clinician participants identified facilitators and barriers to successful PROMs integration into routine CL/P care. Notable barriers included lack of staff time and administrative support to administer and interpret PROMs data.

“I thought it was really hard work, and was quite labor intensive [administering CLEFT-Q].”

“The scale for some questions is not the same scale for other questions. So therefore, you can’t say, oh, they’re all fives, they’re doing really well. Are they’re all zeros, they’re not doing well … whereas our smiley faces we look at a piece of paper and within five seconds of looking at it, we have enough of an understanding of where things are at.”

Some clinicians commented on the length of time it took patients to complete PROMs and the potential burden to the patient.

“I think that we just want to reduce the patient burden. We don’t want them to come to a clinic and be like, right, okay, before you get to see them, you’ve got to fill out this 10-page document.”

Key recommendations from all clinician participants to support PROM use included ensuring appropriate provision of administrative, cleft coordinator, clinical nurse specialist, and psychological support.

“I would say that [reviewing PROMs data] was more of a CNS [Clinical Nurse Specialist] role because somebody could hold that data and follow that data and ensure that services were put in place to help with it. A CNS would be perfect if we could get one of those.”

Some clinicians discussed the need for effective IT systems to store and automatically process PROMs data to make the collected PROM data clinically useful.

“We are very limited by the low IT version that we have … if we could get that information in and the computer crunched the numbers and flagged what we needed to know, then we could use that … if you think 15 min appointments, how long are you going to stare at a piece of paper for rather than talking to your patient?”

Selecting PROMs that are quick to administer and complete, as well as utilizing the same PROM nationally were seen as strategies which would reduce patient burden and enable alignment of practices between CL/P units.

“If we’re not actually collecting the same data in the same way at the same time, then we don’t know where we are as a service and what our patient outcomes actually are compared to others. So yeah, I do think it's important [that teams use the same PROM measure], I think it can be a really good thing. But I think we’re in such the early stages of it, that we need more teams to start using it.”

## Discussion

### Key Findings

The overarching aim of this study was to pilot routine introduction of PROMs in New Zealand CL/P teams. Taken together, the quantitative patient-reported data gives a baseline overview of patient perception of functioning, and the clinician-reported data highlights the merits and considerations of routinely using PROMs within clinical practice. Patient-reported data demonstrated that speech function within the studied cohort was similar to the normative data, this is likely due to the studied cohort having had access to CL/P-related speech language therapy, as is the case for those in the normative sample. The studied cohort reported poorer psychological functioning than the normative group, while clinicians also commented on the lack of psychological support services. This lack of routine CL/P-related clinical psychology services in NZ may explain the lower scores as some of the countries included within the normative dataset do have routine psychology provision. Poorer psychological functioning was particularly pronounced in the adult (18+) age group who scored significantly lower than their younger counterparts which could be indicative of unresolved psychological need in adulthood; a finding supported in existing international literature.^21,31,32^ Interestingly, despite a lack of access to psychological support, speech distress scores were better with the cohort outperforming the normative sample by a significant margin. Possible reasons for this could be that satisfaction with speech outcomes is leading to less speech-related distress, that speech language therapists are taking on an informal counselling role in the absence of formal psychological services (as reported by clinicians in previous studies),^20,33^ or that there are comparably high levels of speech distress within the normative sample due to its heterogeneity. In any case, this patient-reported finding contrasts with caregiver-reported findings from the existing self-developed PROM in use in one NZ CL/P team, which had found speech to be among the most prevalent concerns of caregivers.^24^ This could indicate a difference in the validity of the measures, a difference between patient and caregiver perspectives, or both.

It was anticipated that routine collection of PROM data would lead to treatment plans and consultations being more patient-centered and informed due to being able to use PROM data to guide decisions. However, clinician interviews revealed that none of the CL/P teams used the CLEFT-Q data available to them within their clinical practice citing time constraints, therefore it was not possible to observe any effect. However, clinicians mused that being more confident of patient wishes would be a benefit from PROM usage if teams were sufficiently resourced to use PROMs routinely. From their involvement with collecting PROMs data, clinicians identified some key facilitators and barriers to success with routine PROMs use, the most prominent being around minimizing the amount of time that PROM administration and interpretation take clinicians and patients, and that introduction of PROMs is supported by adequate administrative and logistical support.

### Cleft Type, Ethnicity, Age, and Gender Differences

No differences by cleft type or ethnicity were found on the measures analyzed. A lack of differences by cleft type is also observed in the CLEFT-Q normative data (normative data by ethnicity is not available for the CLEFT-Q).^28^ However, it was found that those over the age of 18 reported poorer psychological function than those under the age of 18. This variance in psychological function by age in this NZ sample was much more pronounced than smaller age variances reported in the normative data. Similarly, on the normative data, there was little variation on social function by gender, however this study found females reported poorer scores in this area than males. This finding is aligned with general studies in adolescent and adult mental health which have found females to have poorer average measures of mental health than males across multiple countries,^34^ including on self-reported data in New Zealand.^35^ The intersectionality of CL/P, gender, and age suggest additional psychological supports for more vulnerable groups should be explored.

### Staffing and Technology

Throughout the implementation of this study, there has been some resistance to introducing PROMs into CL/P teams in NZ, a finding evidenced by clinician reports within this study. While some of this resistance appears to be a general skepticism toward external audit, most commonly, the concerns regarded a perception that PROMs would increase workload. Such findings have also been reported in previous studies.^7,36^ There is evidence to suggest that when implemented routinely and effectively, PROMs have the potential to save time and reduce patient burden by taking the guesswork out of consultations.^5,37^ However, to establish an effective system requires adequate staffing. The lack of administrative support was widely discussed, with administering and interpreting PROMs not currently within any team member's job description. Despite the CLEFT-Q data being made immediately available online to CL/P teams as part of this pilot, no teams used the collected data to inform their patients’ clinical care. Clinicians reported difficulties accessing the data stating that many aspects of CL/P care remain paper-based due to outdated IT systems. For PROMs to be used to their full potential, efficient, automated IT systems which automatically score data, add to the patient's record, and flag areas worthy of follow-up to the clinician are useful supports.^10,36^ Indeed, the need for health service investment in IT systems to support efficient, automated interpretation of PROMs throughout New Zealand and Australia has already been identified.^10^ Computerized administration and scoring of PROM data has already been demonstrated successfully within orthopaedics,^38^ and the benefits of clearly and efficiently presenting PROM results to patients and clinicians alike using pie charts and bar charts has been demonstrated within cancer care.^8^ Although PROMs admin tasks currently require human intervention, with the advancement of artificial intelligence technologies, it is anticipated that these tasks can be supported by automation.

### Balancing Clinic Pressures with PROM Validity

Several clinicians reported their team's preference for using unvalidated self-developed questionnaires as PROMs, citing speed and ease of administration and interpretation as key benefits of their use. While this may offer some advantages in the clinical setting, it does not allow for comparison against other populations which limits the utility of the data the PROM can provide.^39^ While teams are to be commended for incorporating their own PROM data into clinical care and recognizing the importance of patient voice, to ensure that PROM data is psychometrically valid, it is best practice to use existing, standardized, validated PROMs; a recommendation made previously by the Health Services Research Association of Australia and New Zealand.^10^

Computerized-adaptive testing (CAT) may offer a practical solution which balances time pressures with the benefits of using a standardized measure, and CAT has already been developed and trialed successfully for the CLEFT-Q.^40^ CAT employs an algorithm to intuitively choose which CLEFT-Q item to present next to the patient based on their response to the previous question, thereby reducing the total number of questions presented to the patient to achieve a comparable score to completing the entire scale. Use of CAT scales reduces patient burden, and the time required to complete, and score, standardized measures. With measures being quicker to administer, it becomes possible to implement them more regularly and routinely, without resorting to using unvalidated measures for expediency.

CL/P teams in NZ plan to begin collecting ICHOM data from patients for audit purposes, although there is no intention to use the collected PROM data to inform clinical care. It is hoped this decision can be reviewed, as the collection of ICHOM presents a valuable opportunity to also use the collected standardized, validated data within clinical care without increasing patient burden.

### Managing Concern About Impact of Completing PROMs on Patient Wellbeing

Throughout the PROMs trial, there was concern from some CL/P team staff about the impact that completing PROMs could have on participants’ emotional wellbeing; specifically that by completing PROMs, patients could experience elevated rates of distress, and that the PROMs would develop issues for patients which weren’t there previously. While understandable, existing evidence within general mental health and suicidal ideation literature does not suggest that discussing an issue creates the issue, and by extension, avoiding a topic of distress does not result in absence of distress.^41–43^ Furthermore, a previous study has specifically investigated the impact of completing the CLEFT-Q scales on the wellbeing of children and young adults^44^ and reported that most participants found completing the CLEFT-Q to be a positive, often cathartic exercise. Of those who reported a negative impact, this correlated with having reported significantly poor scores overall on the CLEFT-Q scales. Their findings reiterate that PROMs such as the CLEFT-Q highlight distress that is already there, rather than creating it. By bringing such distress to a clinician's attention using the PROM, patients are more likely to then receive appropriate support. Understanding this common concern from clinicians is valuable to be able to provide training and reassurance to staff that the use of PROMs is important to help identify those who require additional support.

### The Need for Psychological Services

The issue remains that whether psychosocial concerns are brought to light through PROMs or any other method, CL/P teams in NZ do not currently have adequate resource to address these. The poorer psychological function outcomes in NZ reported through this study, alongside clinicians’ reports indicate need for provision of psychological services. CL/P can have a multifaceted impact on psychological wellbeing including concerns around appearance and/or speech, challenges with treatment decision making, medical trauma, bullying and discrimination etc.^21,45–47^ These challenges are best supported by a Clinical Psychologist with CL/P-specific expertise (eg, a CL/P team psychologist). Additionally, psychologists are well-placed to administer and interpret PROMs data, so while PROMs can be advantageous and valuable tools to garner patient perspective for all disciplines, these become an even more effective tool when utilized alongside a psychological service.

### Methodological Considerations

The following methodological considerations are acknowledged for interpretation of results and generalizability of these to the larger population.

- Selection bias: CL/P teams reported having been selective about who they advertised the study to depending upon how likely they felt people were to respond. While this approach is pragmatic, it is not aligned with the premise that every patient should have equal opportunities to feedback into their own care and could have introduced bias into the responses. Ideally, PROMs would be administered to everyone and completed either before attending clinic, or on the day of clinic to allow them to have the most clinical utility.
- Influence of parent-report: Although the minimum age of 12 was selected to increase the likelihood that the responses were those of patients, rather than parents, it must be acknowledged that it is likely some patients had parental input when completing the PROMs. This is a limitation of the real-world clinical environment too. A future consideration for clinical practice and research could be to accompany PROMs with parent-reported outcome measures and have a psychologist or social worker facilitate discussion around any discrepancies. Previous research has suggested consulting with adolescents while their parents are out of the room^33^; this could present an opportune time for both parents and patients to complete PROMs.
- Need for greater MDT representation: Within this study, 4 SLTs and 1 Cleft Coordinator were nominated on behalf of their teams to participate in the PROMs pilot. While these representatives offered proxy reporting from their MDT colleagues, greater representation is needed from other disciplines such as surgery and orthodontics to understand additional clinical perspectives of PROMs and to facilitate effective collection of PROM data from other disciplines (eg, measures of appearance satisfaction).
- Limitations of the CLEFT-Q normative data: The published international CL/P normative data for the CLEFT-Q^28^ has a large sample size with geographic, linguistic, age, and gender diversity. However, normative CL/P data is collated from 12 different countries, including some countries with highly resourced CL/P services which include adult care and routine psychological care (eg, UK, Sweden etc), and others from low- and middle-income countries with comparably less resource. A detailed breakdown of the normative data by country is not available within published data. Additionally, while the CLEFT-Q CL/P norm tables do offer a breakdown by cleft-type, age, and gender, there is not reporting by ethnicity. Furthermore, the published normative data for the CLEFT-Q is limited to the normative mean, standard deviation, and *n* figures, rather than the entire dataset. Although the standard deviations would indicate there may be skewness within the normative data (eg, two standard deviations above the mean exceeds the maximum score of 100 indicating a potential ceiling effect), the normative psychometric and validity data^28^ do not indicate any non-normal distribution.

## Recommendations

Several recommendations for clinical practice and service provision are made arising from this study.

- Routine use of PROMs should be incorporated into CL/P clinics as they provide a standardized method to gather individualized information which cannot be obtained from objective assessment, to provide patient-centered care, evaluate change over time for individual patients, and to evaluate the CL/P service.
- Using the same standardized PROM nationally (and ideally, internationally) allows for greater comparison between patients and CL/P teams. Using a PROM which is standardized allows reliable interpretation of patient scores relative to normative data.
- All CL/P teams should have a Cleft Coordinator position (or equivalent), and this position requires sufficient administrative support to oversee initiatives such as PROMs, review patient data, and support the team's participation in research.
- Efficient and automated IT systems (such as computerized adaptive testing) are needed to support PROMs implementation to reduce patient and clinician burden and allow for quick scoring and retrieval of assessment scores which inform consultations, and are linked to the patient's record.
- Clinical Psychology services should be implemented to address unmet psychological need within the CL/P population, including those aged 18+. The CL/P team psychologist is an important liaison between patients and the rest of the team and contributes to CL/P teams in a multitude of ways making them an essential part of the team.^48^ Having psychology provision would provide the rest of the CL/P team with a pathway for escalation of any psychological concerns identified within PROMs or elsewhere.

Additionally, there are recommendations for future research.

- It is recommended that a future study examines patient perspectives of PROM administration into NZ CL/P teams. Patient voice should be considered when selecting a suitable PROM to ensure that patients feel the measure adequately represents their views and is not overly burdensome to complete.
- Collect comparative data from other disciplines. This study originally sought to also seek CLEFT-Q appearance measures from patients engaged with orthodontic services. However, at the completion of the study period, only 13 participants had provided appearance data; a dataset which was too small to analyze meaningfully. Collaboration with orthodontic and surgical colleagues is integral to ensuring data across all disciplines engaged with cleft care is understood.
- Expansion of CLEFT-Q normative data. Current normative data of the CLEFT-Q is based on participants aged 8–29. CL/P is increasingly viewed as a lifelong condition, and adult returners are likely to make up a larger proportion of patients over time; it is important that patient perspective of those aged 30+ is also collected and represented. Furthermore, cultural and healthcare provision influences will likely impact responses to the CLEFT-Q questions, it would be useful to be able to select additional demographics such as country, ethnicity and language for comparison to the studied cohort. Is recommended that a future study examines patient perspectives of PROM administration into NZ CL/P teams. Patient voice should be considered when selecting a suitable PROM to ensure that patients feel the measure adequately represents their views and is not overly burdensome to complete.

## Conclusion

While NZ adolescents and adults with CL/P report similar speech and social function, and better speech distress and school function scores than international CL/P norms, reductions in psychological function highlight a substantial gap in psychological support for people with CL/P in NZ. Routine, consistent provision of psychology services for people with CL/P is warranted, in line with international standards of care. Automated IT workflows, coupled with sufficient Cleft Coordinator provision, and multidisciplinary usage of standardized, validated PROMs are pivotal to their routine implementation.

## Supplemental Material

sj-docx-1-cpc-10.1177_10556656261431254 - Supplemental material for Piloting Patient Reported Outcome Measures in Cleft Lip and Palate Services in Aotearoa New Zealand Supplemental material, sj-docx-1-cpc-10.1177_10556656261431254 for Piloting Patient Reported Outcome Measures in Cleft Lip and Palate Services in Aotearoa New Zealand by Kenny Ardouin, Nicola Stock, Tika Ormond and Phoebe Macrae in The Cleft Palate Craniofacial Journal
