# Supplementary material for: Piloting Patient Reported Outcome Measures in Cleft Lip and Palate Services in Aotearoa New Zealand
Source: Cleft Palate Craniofac J. 2026 Apr 15;63(11):3805–16. doi: 10.1177/10556656261431254 (PMC13646430; doi:10.1177/10556656261431254)
Supplement: Supplementary file 1 [file sj-docx-1-cpc-10.1177_10556656261431254.docx]

**Supplementary Materials**

**Table A**

**Demographic Information (requested of all patient participants)**

- Age (12-17, 18-29, 30+)
- Gender (Female, Male, In another way)
- Ethnicity (free text)
- Cleft type (all options presented)
- Region^

^ *Region data was collected based on recruitment tracking methods. Participants were not directly asked their region.*

**Table B**

**Clinician Exit Interview Questions**

***Questions***

1) What existing strategies did your team have in place before participating in the patient reported outcome measures trial to incorporate patient voice into clinical decision making?

2) How have you found adding several of the CLEFT-Q scales to some of your appointments as part of the patient-reported outcome measures trial?

3) What do you see as the benefits of using patient-reported outcome measures (PROMs) clinically?

4) What do you see as the barriers to implementing PROMs within everyday cleft care on an ongoing basis?

5) Is the information obtained on the PROMs useful to you clinically?

*Follow up prompt question:* Are you likely to discover something about your patients that you otherwise would not know if it weren’t for them completing the PROMs?

6) Do you feel that it would be important for teams across New Zealand to be using the same patient reported outcome measures to allow for comparison between services?

*Follow up prompt question:* Do you have a preference for which patient reported outcome measures should be used?

7) Where do you think would be the key points in the cleft treatment journey to implement PROMs?

8) How would you feel knowing that your patients were going to complete a pre and post treatment PROM?

9) Do you think your colleagues are likely to adopt PROMs?

10) How did you find your involvement within this study?

*Follow up prompt question:* Did you feel that you were able to contribute?

**Table C1**

***Summary of Independent-Samples Kruskal Wallis Testing p values by demographic and CLEFT-Q measure (Within Group Differences)***

|  | **Speech Function** | **Speech Distress** | **Psychological Function** | **Social Function** | **School Function** |
| --- | --- | --- | --- | --- | --- |
| **Cleft Type** | *p* = 0.311 | *p* = 0.851 | *p* = 0.740 | *p* = 0.680 | *p* = 0.713 |
| **Ethnicity** | *p* = 0.066 | *p* = 0.424 | *p* = 0.749 | *p* = 0.909 | *p* = 0.461 |

* *indicates that finding is statistically significant (p < 0.05)*

**Table C2**

***Summary of Independent-Samples Mann-Whitney U Testing p values by demographic and CLEFT-Q measure (Within Group Differences)***

|  | **Speech Function** | **Speech Distress** | **Psychological Function** | **Social Function** | **School Function** |
| --- | --- | --- | --- | --- | --- |
| **Age** | *p* = 0.458 | *p* = 0.321 | *p* = 0.030* | *p* = 0.040* | *p* = 0.177 |
| **Gender** | *p* = 0.523 | *p* = 0.329 | *p* = 0.369 | *p* = 0.050* | *p* = 0.088 |

* *indicates that finding is statistically significant (p < 0.05)*

**Table D**

***Thematic Analysis of Clinician Interviews***

| ***Theme one: Pre-existing attitudes and practices to incorporating patient perspective within cleft care*** | |
| --- | --- |
| **Code** | **Exemplar Quotes** |
| Existing practices which incorporate patient voice within clinic | “We had nothing prior to this.”  “I haven't really used them [PROMs] much in clinic…but I’ve seen the ICHOMs…we use our own speech ones with the RhinoCleft® acceptability and intelligibility as they’re patient reported.”  “We have our smiley faces questionnaire which has 10 or so questions with smiley face scale to say what people think of smile, face, social interactions, various things…that gives us a bit of an idea of what are the things that they feel need to be addressed.”  “Discussion in the clinic would be the usual way… their [patient] input would be sought in that way. I can't think of any other way that it [patient voice] currently happens.” |
| Cleft team professionals’ perspectives towards PROM trial and implementation | “I think if we can get into a good system of doing it [PROMs] in advance…which I think we certainly could, I think it would be really useful actually.”  “[We should administer PROMs] at every intervention, really all of the time… continuous and frequent.”  “A general culture within a team or an attitude shift needs to occur…no one likes audits…but I think outcome measures are so important. And I think we need them all the time.”  “I was very enthusiastic at the beginning. I couldn't believe how unenthusiastic my team were…I think this research here is very needed.” |
| Reflections on participating in the PROMs trial | “I'm quite proud…I just jostled their [other clinicians’] cage and said, Come on, you know, this is really important.”  “We really need to do this. We did. Very pleased.”  “It's [using the CLEFT-Q PROMs] not changed anything that we've done at appointments at all. Patients did your questionnaire, your CLEFT-Q, completely separate to anything that they gave us…it didn't actually change anything about what we saw, or did, or acted on in clinic.”  “Our smiley faces works well for us. And we will continue to use them until we find another way of finding getting that easy access to information at the time. It might not be the best thing but it works.”  “I think that the one we just used [CLEFT-Q] seemed pretty sensible to me, I think that covered a lot of areas quite quickly… it's obviously that weigh up between it being getting the information you need in a way that's not too much of a time commitment to put people off.” |
| ***Theme Two: Perceived influence of routine PROMs use within cleft clinics*** | |
| **Code** | **Exemplar Quotes** |
| Anticipated benefits of using PROMs | “There were a couple that reported [low] scores, who I thought their speech was just about completely on target… I wondered whether the tool would be a way to just have a conversation about that…because from our assessment we wouldn't get that information.”  “It [PROMs] would mean that a clinician has a little bit more insight than they might otherwise…I'm very aware that sometimes you get in a room of clinicians and you go, oh, I can't remember anything that I was going to ask!”  “It could help to inform for the child, the reason why the parent keeps dragging the child to the appointment all the time as well… Why do we care? Why do we want you to keep coming back? Why do we want to help you? Do you think you need help?”  “Those reported measures can help us to understand whether or not something does need to be ‘fixed’ because we as professionals think it needs to be versus whether the patient and/or family think it needs to be.”  “Does it [use of PROMs] support equality of care, because we're ensuring that the same questions are asked to everyone? Regardless, who walks in the room, when, by human nature, we do ask different things to different people just naturally due to their conversation skills or their culture…it's a bit more sort of standardization of questions.”  “Perhaps they might get more time to consider a more sort of considered and honest answer to things [when using PROMs]. Whereas in the middle of an appointment where there's lots of professionals in the room, and they're asked outright, something, they might not give themselves the time to answer as deeply as they want. Or perhaps they fear taking up too much time or not saying the right thing.”  “I think it will be a really helpful thing to be able to get their patient voice and get what's on top for them, because it might not be what we're looking at. It might be something so irrelevant.” |
| Potential concerns arising from using PROMs routinely | “If we have used the patient outcomes to measure how we're doing it's just what they feel and what they think their appearance is like…we're getting them to highlight problems when there really isn't a problem. That is one of the reasons that they [colleagues] have told us that they don't think patients should be reporting outcomes.”  “The concern [with using PROMs clinically] is that it might bring hope to people that something can be fixed, when in fact, the surgeon knows that due to surgical limitations it can't be.”  “The treatment is complete, but yes, it might not be perfect…Is that enough for that person, but they're finishing treatment, because that is the best the clinician can do…you could do a PROM after treatment, but the only person who could be of benefit at that point is a psychologist, because the orthodontist is done. And if you don't have a psychologist to then talk about how that person feels about that…for the record, we would love a clinical psychologist as part of our team.” |
| ***Theme Three: Facilitators and barriers to introducing PROMs routinely on cleft teams*** | |
| **Code** | **Exemplar Quotes** |
| Barriers to routine PROM use within clinical care | “[It was] really hard [to implement PROMs within clinical appointments]. Not really possible. We started off trying to do it but it was just too clunky…we had to leave being with a patient…we often are spread quite thin in those clinics.”  “I would say that [reviewing PROMs data] was more of a CNS [Clinical Nurse Specialist] role because somebody could hold that data and follow that data and ensure that services were put in place to help with it. A CNS would be perfect if we could get one of those.”  “I thought it was really hard work, and was quite labour intensive [administering CLEFT-Q].”  “The scale for some questions is not the same scale for other questions. So therefore, you can't have a you can't say, oh, they're all fives, they're doing really well. Are they're all zeros, they're not doing well… whereas our smiley faces we look at a piece of paper and within five seconds of looking at it, we have enough of an understanding of where things are at.”  “It comes down to admin time…we have started doing ICHOM. So we are actually using a lot of those CLEFT-Q scales anyway. And the admin of that falls to me because I don't have admin support…we're still doing on paper, IT is possibly further down the track, but not now.”  “we are very limited by the low IT version that we have…if we could get that information in and the computer crunched the numbers and flagged what we needed to know, then we could use that...if you think 15 minute appointments, how long are you going to stare at a piece of paper for rather than talking to your patient?”  “it's [PROMs] not usable data, unless someone deals with that information. And we don't have somebody to deal with that information.” |
| Considerations for successful implementation of PROMS | “I think, [centre] really, really could do with a clinical nurse specialist, somebody who could, you know, coordinate these kind of things [holistic care].”  “I don't know if the tool itself really matters, so long as the data is being collected.”  “I think that we just want to reduce the patient burden. We don't want them to come to a clinic and be like, right, okay, before you get to see them, you've got to fill out this 10 page document.”  “I think I would quite like to have these reported measures, before and after sort of a significant block of therapy, to help inform what the goals of therapy are so that we aren't the ones instigating and creating the goals, but rather, we are creating goals that are specific for what the family want. And then at the end of the therapy, ensuring how far we come to achieving that goal?”  “If we're not actually collecting the same data in the same way at the same time, then we don't know where we are as a service and what our patient outcomes actually are compared to others. So yeah, I do think it's important [that teams use the same PROM measure], I think it can be a really good thing. But I think we're in such the early stages of it, that we need more teams to start using it.”  “I guess it wouldn't be vital [for all teams to use the same PROM]. But it would make sense because that would provide the opportunity to have a lot of information, which potentially could help inform services…I think using the same tool would make sense if possible…unless it was too logistically difficult.” |
